# Epidemiology of developmental dysplasia of the hip within the UK: refining the risk factors

**DOI:** 10.1007/s11832-016-0798-5

**Published:** 2016-11-19

**Authors:** Timothy Woodacre, Thomas Ball, Peter Cox

**Affiliations:** PEOC, Royal Devon and Exeter Hospital, Exeter, Devon EX25DW England, UK

**Keywords:** DDH, Epidemiology, New risk factors

## Abstract

**Purpose:**

The epidemiology and risk factors for developmental dysplasia of the hip (DDH) are still being refined. We investigated the local epidemiology of DDH in order to define incidence, identify risk factors, and refine our policy on selective ultrasound screening.

**Methods:**

With a cohort study design, data were prospectively recorded on all live births in our region from January 1998 to December 2008. We compared data on babies treated for DDH with data for all other children. Crude odds ratios (ORs) were calculated to identify potential risk factors. Logistic regression was then used to control for interactions between variables.

**Results:**

There were 182 children born with DDH (with a total of 245 dysplastic hips) and 37,051 without. The incidence was 4.9 per 1000 live births. Female sex (adjusted OR 7.2, 95% confidence interval [CI] 4.6–11.2), breech presentation (adjusted OR 24.3, 95% CI 13.1–44.9), positive family history (adjusted OR 15.9, 95% CI 11.0–22.9) and first or second pregnancy (adjusted OR 1.8, 95% CI 1.5–2.3) were confirmed as risk factors (*p* < 0.001). In addition, there was an increased risk with vaginal delivery (adjusted OR 2.7, 1.6–4.5, *p* < 0.001) and post-maturity (OR 1.7, 1.2–2.4, *p* < 0.002).

**Conclusions:**

One in 200 children born within our region requires treatment for DDH. Using both established and novel risk factors, we can potentially calculate an individual child’s risk. Our findings may contribute to the debate regarding selective versus universal ultrasound screening.

**Level of Evidence:**

Prognostic Study: Level 1.

## Background

Developmental dysplasia of the hip (DDH) is a common and preventable cause of childhood disability, and forms a large portion of paediatric orthopaedic practice. It is generally agreed that late diagnosis (often quoted as diagnosis after 3 months) leads to a higher chance of needing surgery and a higher risk of long-term complications [1].

The epidemiology and risk factors for DDH are still being refined. The incidence in the UK before ultrasound (US) screening became available was quoted as 1–2 per 1000. Since the advent of selective US screening, which selectively ultrasounds the hips of babies who are thought to be at high risk of DDH, estimates of the UK incidence have increased and range from 5−30 per 1000 [2].

There is consensus that a family history of DDH, breech presentation, female sex, primiparity and oligohydramnios increases the risk of a baby having DDH, although only babies in the first two categories are generally selected for US screening [3]. There is inconsistency in the literature as to whether the risk is affected by birth weight, prematurity, multiple pregnancy, mode of delivery or the presence of foot deformity [4–6].

We therefore examined the epidemiology of DDH in our region, with particular attention to risk factors.

## Methods

The catchment region for our centre has a population of 450,000. We analysed this region from January 1998 to December 2008. The annual birth rate over this period was 3500 per year, with 37,233 live births recorded.

We examined data, prospectively recorded by the senior author, on all referrals to the regional hip screening clinic over this 11-year period. The data gathered included demographic variables, family history, intra-uterine position, method of delivery, gestational age at birth, birth weight, presence of other deformities, oligohydramnios and other gestational and medical history.

The relevant control group consisted of all other live births (37,051) in the same region over the same period. Birth data for the control group were collected prospectively by midwives and recorded on the local Liveslot database/Stork record. The data collected included all of the variables collected on those babies referred to the DDH clinic, with the exception of oligohydramnios and foot deformities (poorly documented in the control group).

### Diagnosis of DDH

The approach to diagnosis of DDH was consistent throughout this period. An obstetric history was taken, including risk factors for DDH, breech presentation and family history. The hips of all the babies were clinically examined by a junior paediatric doctor on the post-natal ward and again at 6 weeks by the general practitioner.

The regional DDH service consisted of a ‘one-stop’ neonatal hip clinic providing repeat clinical examination by an experienced consultant, US and Pavlik harness treatment for babies at risk. If the post-natal examination was abnormal, babies were referred immediately and seen and scanned at two weeks after birth. If the examination was normal but babies had a risk factor, they were seen and scanned at 5–6 weeks. If the 6-week examination by the GP was abnormal they were seen within 2 weeks of referral (7–8 weeks after birth). Treatment was decided on the basis of a combination of Graf grading on a coronal US view, and instability on dynamic anterior US [7, 8]. Babies with Graf 4 hips were placed immediately in a Pavlik harness and followed up with serial US, as were most Graf 3 hips. Graf 2 hips and Graf 3 hips with minimal decentering were monitored with serial US, with the majority spontaneously improving. Between 6 and 12 weeks, hips with persisting grade 2c dysplasia or instability were treated in harness. At 12 weeks, any baby with persisting dysplasia of grade 2b was treated. Treatment was escalated as appropriate based on response to Pavlik harness management. Whether treated or not, babies with hips of grade ≥2a were followed up with US scanning until normality on US was reached. Radiographs were performed at 1 year for any baby failing/requiring a greater level of intervention than Pavlik harness management. Follow-up was only discontinued if they were normal.

### Case definition

Case definition included all decentered or dislocated hips (Graf III and IV), critical range dysplasia (Graf IIc hips) that persisted beyond 6 weeks of age, and instability diagnosed on US that persisted beyond 6 weeks. ‘Late diagnosis’ was defined as babies presenting after 12 weeks, in common with several other authors.

### Exclusions

Babies with teratological and neuromuscular dysplasia of the hip were excluded. Babies reviewed at the clinic who were not born within the regional catchment area were excluded. Six babies had little or no data recorded from the referral clinic and were therefore also excluded.

### Data handling

The control group data was imported into a spreadsheet and validated. Births from out of the region were removed. Duplicate data was removed. Any missing data was quantified and extraneous data removed. As the DDH group contained no twins, multiple births were also removed from the control data.

Free text entries recorded under ‘family history’ box were scrutinised. Items such as ‘CDH’, ‘DDH’, ‘clicky hip’, ‘hip dysplasia’, ‘hip dislocation’ and ‘treatment for infant hip problems’ were given a positive value. Family history of Perthes disease, slipped upper femoral epiphysis, hip fractures or infection/septic arthritis of the hip were given a negative value.

Maternal age was stratified into groups of 5 years and gestational age was counted in weeks, and counted as ‘post-maturity’ if >38 weeks; all other continuous variables were treated as such.

Intra-uterine position was recorded at onset of labour and classified as cephalic, breech or ‘other’, which included transverse position and shoulder dystocia.

The known cases of DDH were found and removed from the control group, so that individuals were not counted twice. The agreement between the data sets was checked (the data relating to babies with suspected DDH recorded by the referral clinic and data recorded on the Liveslot database/Stork record) with particular reference to the potential risk factors. The agreement between the datasets was extremely high, except in family history, where there was a 56% agreement. We therefore used family history data from the Liveslot database, so any recall bias in the DDH study group was eliminated.

### Statistical analysis

Data were analysed using SPSS. For each variable we performed univariate analysis, looking at crude odds ratios (ORs) for DDH, and calculated 95% confidence intervals and *p* values. Variables with crude ORs showing significance (*p* < 0.05) or borderline significance (0.10 > *p* > 0.05) were considered potential risk factors, and were then re-analysed using logistic regression to discover and control for interactions and confounding factors.

## Results

**Fig. 1:**
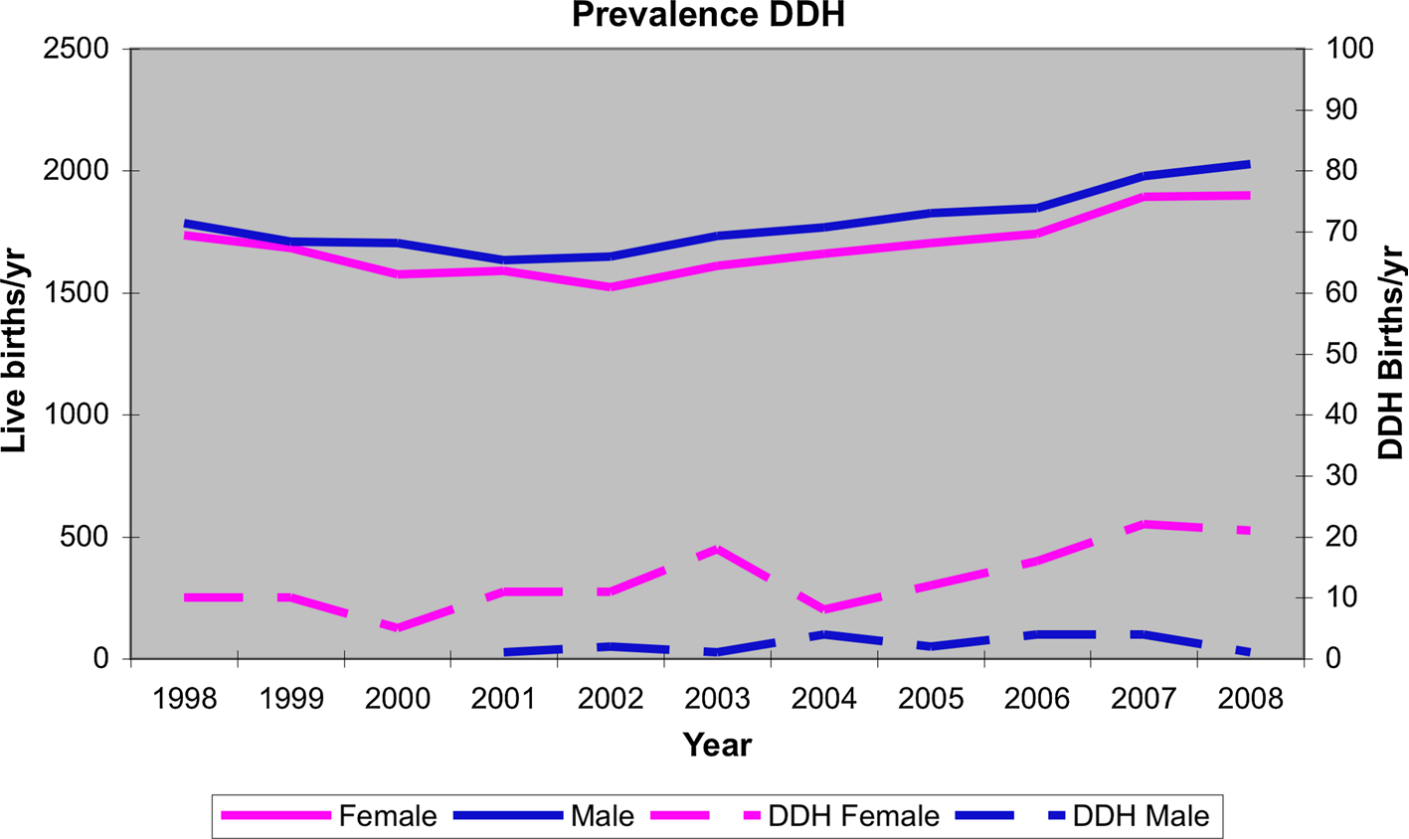
Birth rate and DDH prevalence over the study period

Between 1st January 1998 and 31st December 2008, there were 182 children (245 dysplastic hips) diagnosed with DDH, and 37,051 other singleton live births from within the area. There were 159 females with DDH and 23 males. One hundred and forty-seven children were diagnosed before 12 weeks while 35 presented later than this. Of 147 children presenting before 12 weeks, 133 were successfully managed by Pavlik harness alone. Three late presenters were tested with Pavlik harness management—one was successful while the other required a higher level of intervention [8, 9]. The overall incidence of DDH requiring treatment was therefore 4.9/1000 with a late diagnosis rate of 0.94/1000 (Fig. 1).

Variables with significant association with DDH (*p* < 0.05) were female sex, breech position, ‘other position’ (transverse lie, shoulder dystocia), family history, first pregnancy, second pregnancy, caesarean section, post-term delivery and maternal age 20–24 years (Table 1). Statistically significant variables were subsequently included in logistic regression analysis. Seven risk factors remained significant (Table 2). They are, in order of strength of association:

**Table 1:** Variables with significant association to DDH

Risk factor	Odds ratio (crude)	95% Confidence interval	*p* value
Female sex	7.3	4.5–10.1	0.000
Breech position	8.6	6.1–12.1	0.000
Other position	3.49	0.86–14.22	0.063
Family history	12.6	8.9–17.8	0.000
First pregnancy	3.6	2.3–5.6	0.000
Second pregnancy	2.4	1.49–3.77	0.000
Caesarean delivery	1.72	1.25–2.37	0.001
Post-term delivery	2.32	1.58–3.40	0.000
Maternal age 20–24 years	0.46	0.26–0.82	0.007

**Table 2:** Variables with true significant association with DDH following logistic regression

Risk factor	Odds ratio (adjusted)	95% Confidence interval	*p* value
Female sex	7.2	4.6–11.2	0.000
Breech presentation	24.3	13.1–44.9	0.000
Other presentation	5.0	1.2–20.8	0.027
Family history	15.9	11.0–22.9	0.000
1st/2nd pregnancy	1.8	1.5–2.3	0.000
Vaginal delivery	2.7	1.6–4.5	0.000
Born after 38 weeks	1.7	1.21–2.4	0.002

**Fig. 2:**
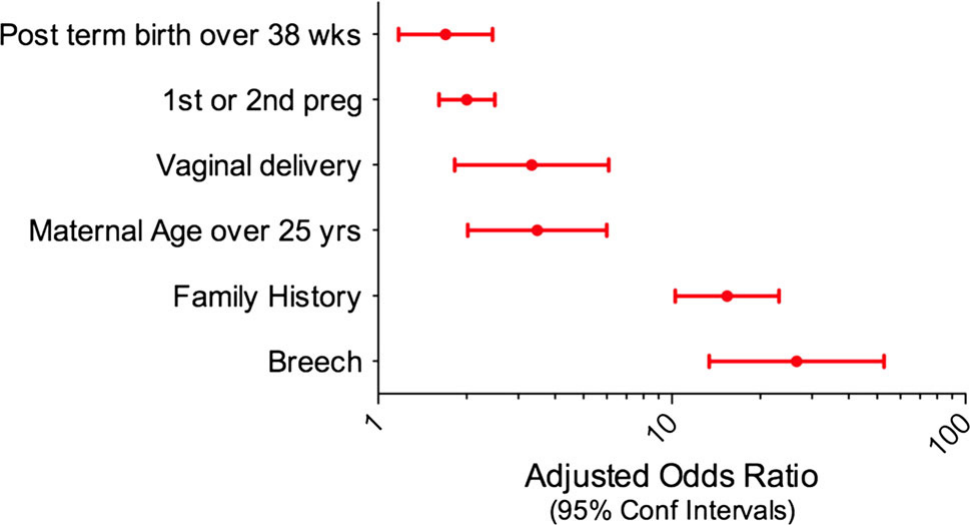
Odds ratio for risk factors for DDH for female children

**Fig. 3:**
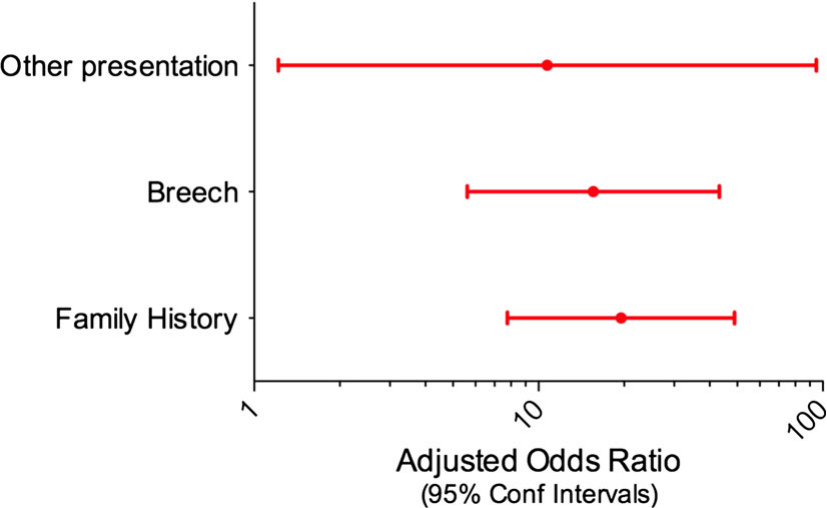
Odds ratio for risk factors for DDH for male children

1. Breech presentation: this increased the risk by an OR of 24.3 (13.1, 44.9).
2. Family history: a positive family history of DDH increased the risk by 15.9, if comparing the more accurate study group data with the control data from the Liveslot database.
3. Female sex: girls were 7.2 (4.6, 11.2) more likely to have DDH than boys.
4. Other presentation: there was a small number of babies in positions other than cephalic or breech, but their OR was 5.0 and this was significant (*p* < 0.027).
5. Vaginal delivery: breech babies are more likely to be born by caesarean section, which explained the high crude OR for caesarean section. Once this confounding was controlled for, caesarean section was found to be protective, and vaginal delivery increased the risk by an OR of 2.7.
6. Parity: there was a decreasing risk of DDH with increasing parity; first and second born children had an OR of 1.8 compared to third and subsequent children.
7. Post-maturity: babies born after 38 weeks were more likely to have DDH (OR 1.7).
8. Several factors showed interaction with the baby’s sex, therefore the above factors were recalculated for female children only. Five of the six variables remained significant (all except ‘other position’) and maternal age over 25 years became significant (Fig. 2). ORs were recalculated for male children, and only three factors remained significant (Fig. 3).

**Fig. 4:**
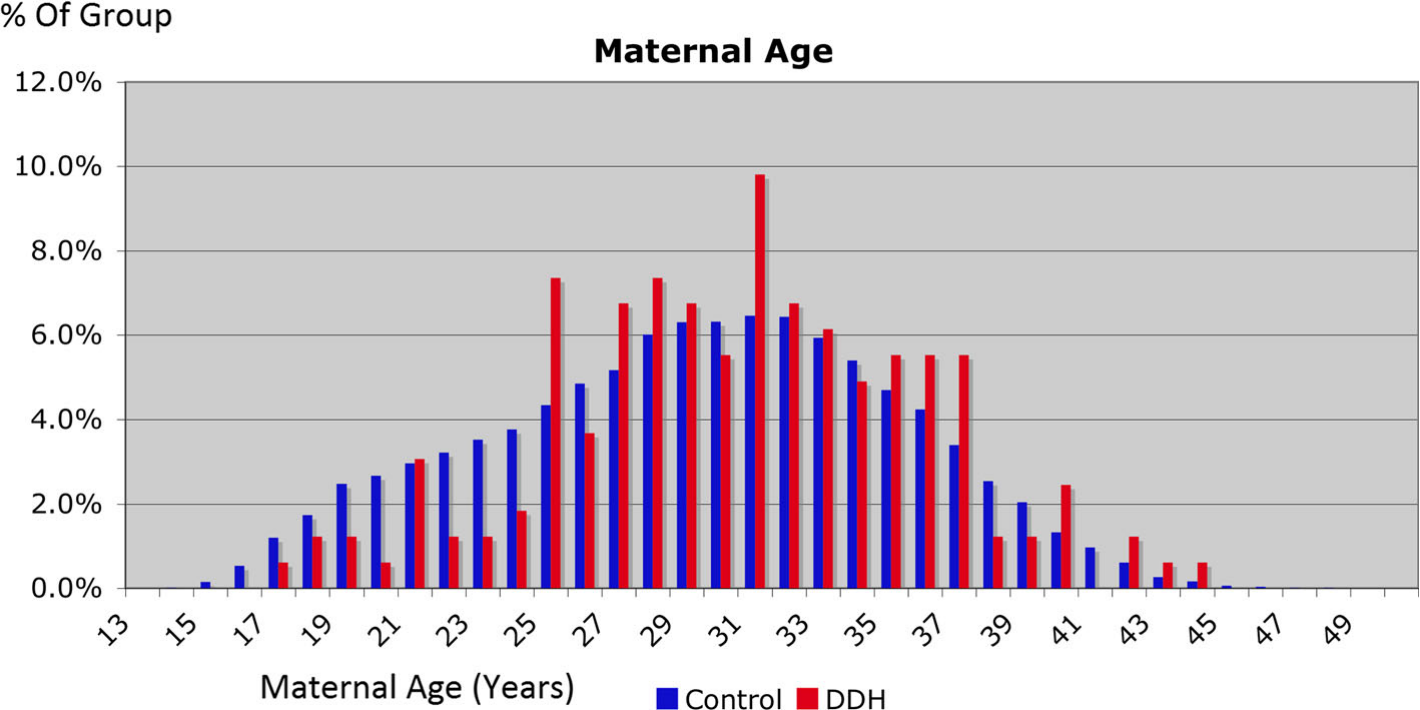
Analysis of DDH incidence relevant to maternal age

**Fig. 5:**
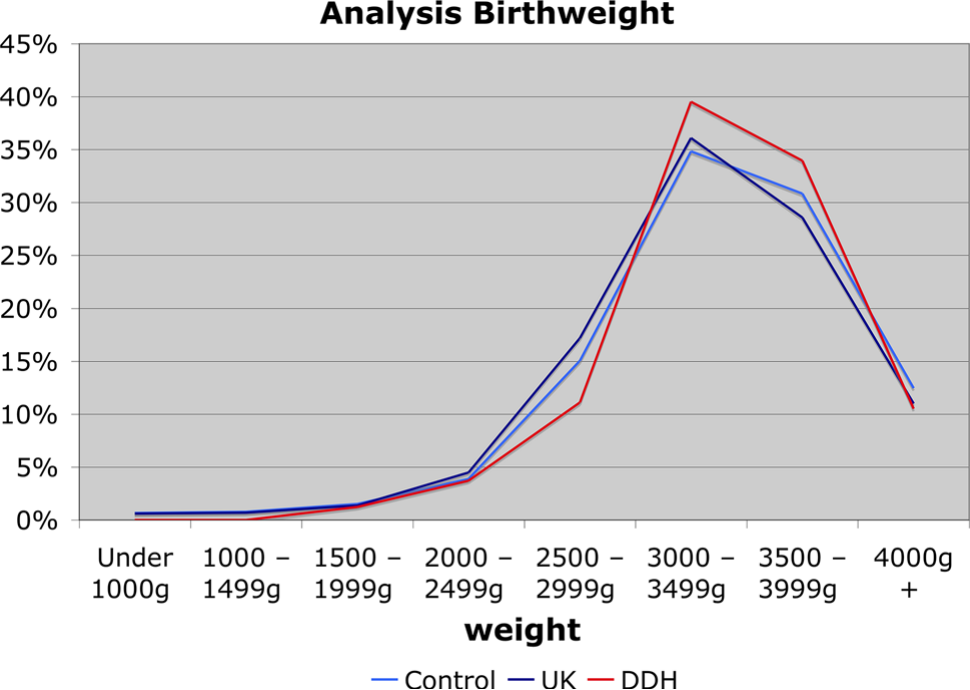
Analysis of DDH incidence relative to birth weight (cases, controls and UK population)

DDH incidence was plotted against maternal age and birth weight, as they are continuous variables and have previously been associated with an effect on DDH incidence [1, 5, 6, 10, 11]. However, the trend in both variables did not reach statistical significance (see Figs. 4, 5).

**Fig. 6:**
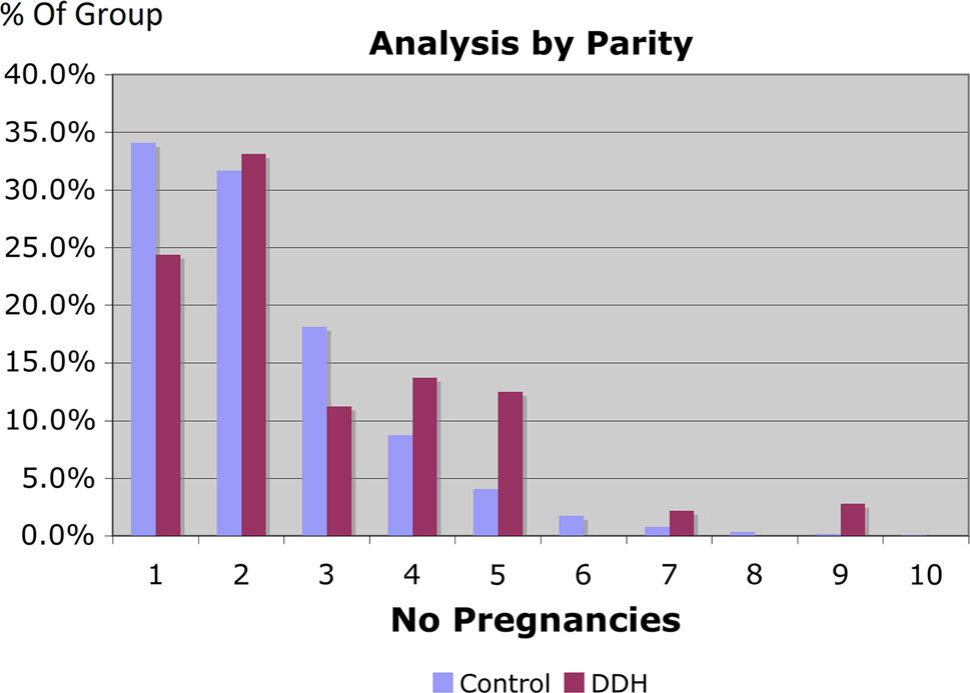
Analysis of DDH incidence relevant to parity

**Fig. 7:**
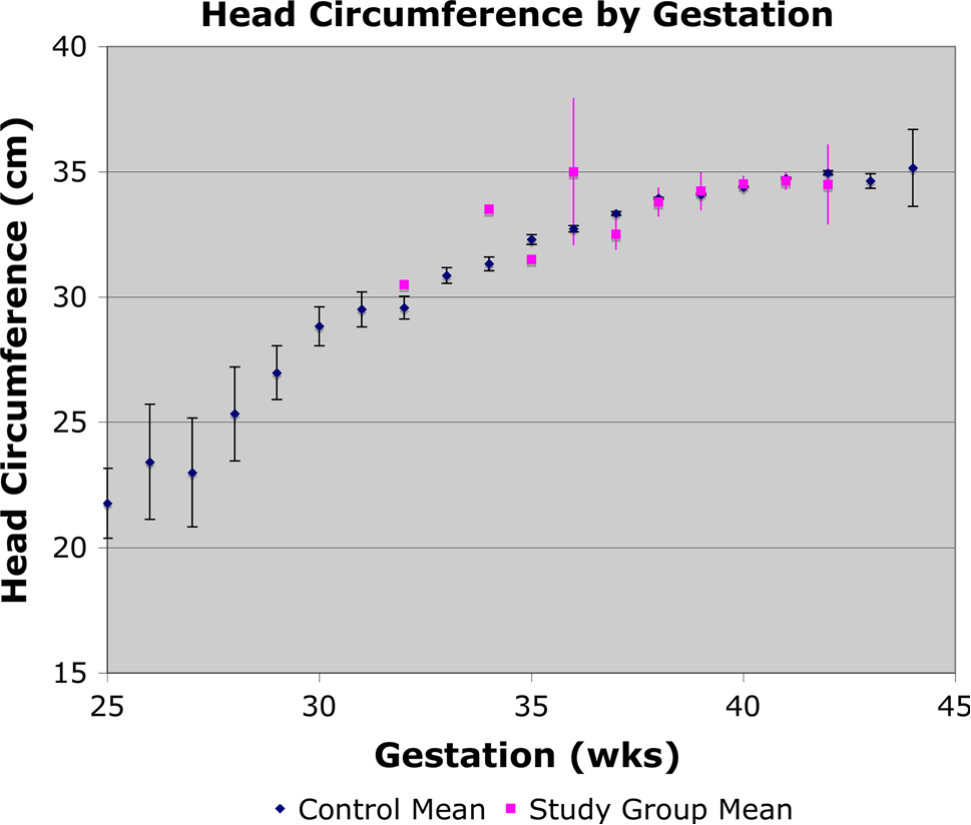
Head circumference relative to gestational age

Within the DDH group a trend was demonstrated towards higher parity (Fig. 6) and higher birth weight (Fig. 5); the control data closely matched UK-wide data. A comparison of head circumference and gestation for the two groups was completed (Fig. 7), demonstrating that the two match closely in both groups and that any effect due to one may be confounded by the other.

**Fig. 8:**
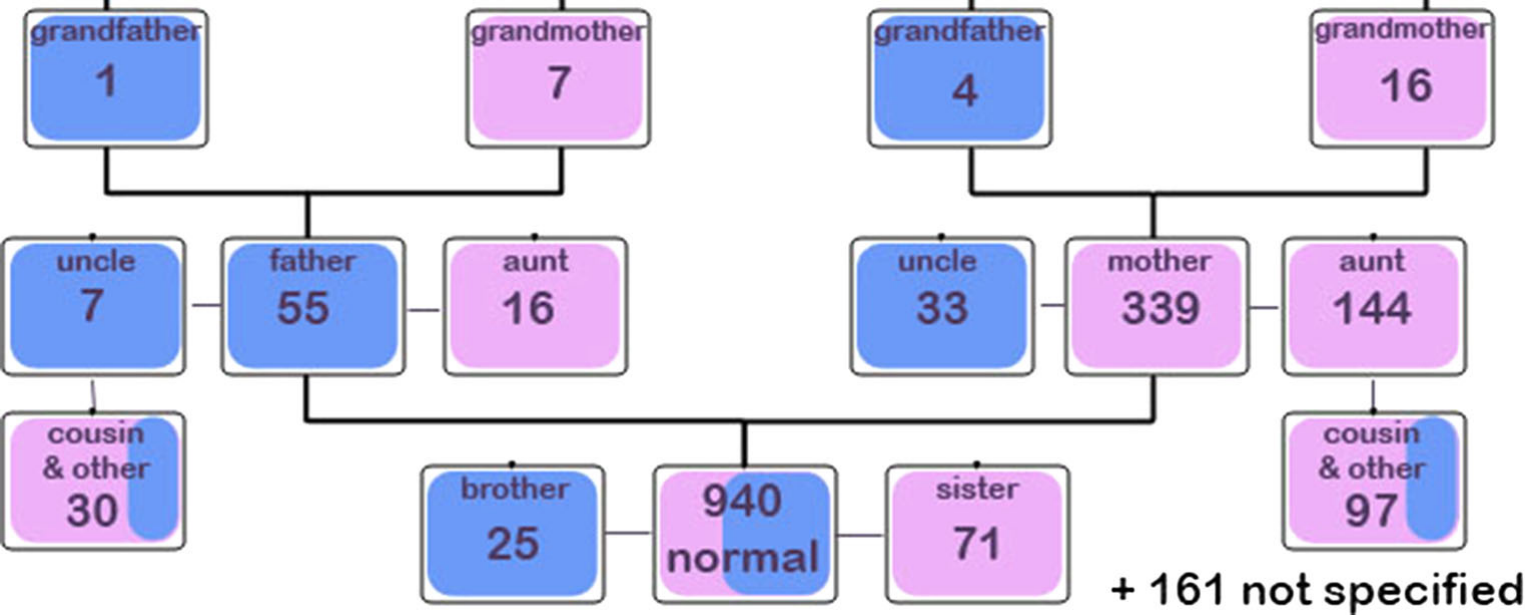
Schematic for relatives of babies from the control group with a positive family history for DDH

### Referral pathway and dysplasia severity

More in-depth analysis was performed on the study group to assess why babies were referred (whether from the neonatal examination, the 6-week examination, later detection or from the presence of risk factors) and whether any pattern existed between referral pathway and severity of dysplasia (e.g., whether there was a greater readiness to diagnose mild DDH in those with risk factors), as this could be seen as evidence of measurement bias. The range of severity of dysplasia was similar for babies referred via all routes, which is against any notion of bias shown to one or other groups (Table 3). A large proportion of those detected clinically had associated risk factors in addition to the abnormal physical findings. Almost all babies sent for US screening had the associated risk factors of breech presentation or a positive family history. A considerable number of babies diagnosed late had risk factors, and should have been referred earlier under our policy of selective US screening.

**Table 3:** Method of referral compared to associated risk factors and severity of dysplasia

Presentation mode	Sex	No.	Positive family history	Breech presentation	Dysplasia (Graf 2a/b/c)	Dislocated (Graf 3/4)
Neonatal examination	Male	12	4	2	1	11
	Female	80	16	24	8	72
6-week check-up	Male	0	0	0	0	0
	Female	10	1	1	0	10
US screening	Male	7	3	3	1	6
	Female	34	15	12	8	26
Late detection	Male	3	1	1	0	3
	Female	36	5	4	3	33

### Family history

**Fig. 9:**
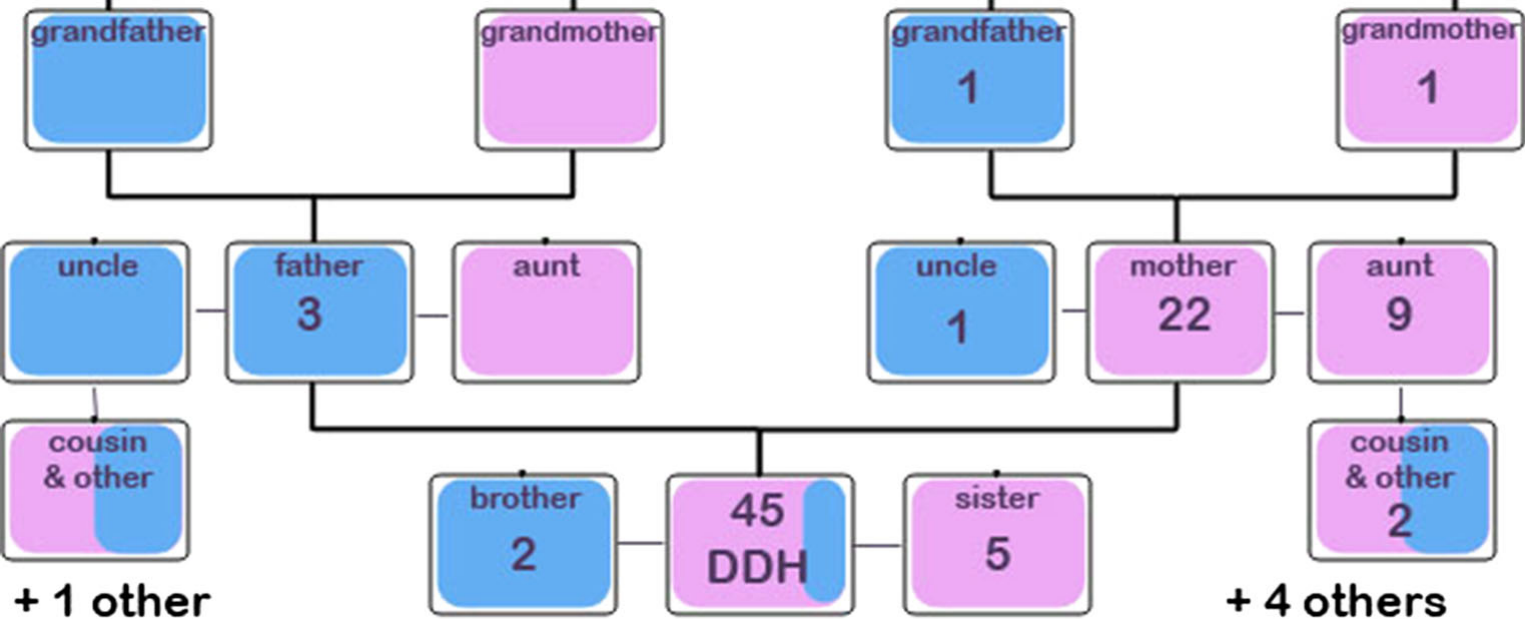
Schematic for relatives of babies from the treatment group with a positive family history for DDH. *Asterisk* the total numbers of each type of relative with DDH in the relevant boxes

Family history was analysed in more detail to ascertain which relatives were most routinely affected (Figs. 8, 9). Nine hundred and forty babies (out of 37,051 total live-births) from the control group (therefore without DDH) had a positive family history suggestive of a relative with hip dysplasia, encompassing 1006 relatives in total. Forty-five babies (out of 182 babies in total) from the treatment group (therefore with DDH) had a positive family history suggestive of a relative with hip dysplasia, encompassing 51 relatives in total.

Tables 4 and 5 present the raw figures and percentages of the occurrence of DDH in relatives subdivided by relative type. An estimate of the likely number of relative types per child was made (assuming mean 2 children per family). In this manner the likely chance of a particular relative type having DDH was calculated for both control and treatment groups. Marked similarity was found in the family pedigree for DDH risk in both treatment and control groups with both demonstrating predilection for female maternal-side relatives and in the following order—(1) mother, (2) sister, (3) maternal aunt, (4) brother, (5) father, and (6) maternal cousin.

**Table 4:** DDH occurrence in relatives of the control group

Type of relative (control data)	No. of children (control data)	Average no. of relative types per child	Estimated no. of relative types in control group	Percentage of relative type with DDH (%)
Mother	339	1	339	33
Maternal aunt	144	1	144	14
Maternal cousin	97	2	48.5	5
Father	55	1	55	5
Sister	50	0.25	200	20
Maternal uncle	33	1	33	3
Paternal cousin	30	2	15	1
Sibling	25	0.5	50	5
Brother	21	0.25	84	8
Maternal grandmother	16	1	16	2
Paternal aunt	16	1	16	2
Paternal grandmother	7	1	7	1
Paternal uncle	7	1	7	1
Maternal grandfather	4	1	4	0
Paternal grandfather	1	1	1	0
Not specified	161			
All relatives		15	1019.5

**Table 5:** DDH occurrence in relatives of the treatment group

Type of relative (of children with DDH)	No. of children (with DDH)	Average no. of relative types per child	Estimated no. of relative types in control group	Percentage of relative type with DDH (%)
Mother	22	1	22	33
Maternal aunt	9	1	9	13
Maternal cousin	4	2	2	3
Father	3	1	3	4
Sister	5	0.25	20	30
Maternal uncle	1	1	1	1
Paternal cousin	1	2	0.5	1
Sibling	0	0.5	0	0
Brother	2	0.25	8	12
Maternal grandmother	1	1	1	1
Paternal aunt	0	1	0	0
Paternal grandmother	0	1	0	0
Paternal uncle	0	1	0	0
Maternal grandfather	1	1	1	1
Paternal grandfather	0	1	0	0
Not specified	5			
All relatives		15	67.5	

### Late presenters

The 34 children presenting ‘late’ (with 36 dysplastic hips in total) had neonatal and 6-week clinical examinations that were thought to be normal. They had no additional risk factors (breech position or positive family history) for DDH that would have warranted an earlier referral.

## Discussion

### Regional incidence, screening and definition of DDH

The incidence of DDH in our region and the rate of Pavlik harness application were within the accepted range [10–12]. Sonographic immaturity [13] and increased soft tissue laxity [14] immediately after birth can lead to over-diagnosis of DDH. This was avoided in our study by DDH only being diagnosed in babies with Graf 3 and 4 hips, or in babies with persistent dysplasia and/or instability after 6 weeks [1].

### Known risk factors

Our study confirmed that female sex, family history of DDH, breech position and primiparity are major contributors to a baby’s risk of developing DDH. Moreover, it quantified the odds of developing DDH if a baby is in possession of risk factors, albeit with relatively wide confidence intervals. This provides the potential to model a baby’s risk of DDH based on the presence or absence of these factors. Although oligohydramnios was not reliably recorded in the control dataset, among the cases, the proportion with oligohydramnios was reliably recorded, but the control data do not allow us to comment on it as a risk factor.

There is potential for measurement bias in our study because those babies with a positive family history or in breech position were routinely screened with US, as opposed to those without. Thus, babies without these risk factors and with only borderline/potentially low Graf-scoring dysplasia that did not result in obvious clinically detectable abnormalities, but which may have caused persistent dysplasia (and subsequent later hip pathology such as osteoarthritis), may have been missed. However, the number of borderline cases was relatively small compared to the number of more severe cases that should, if not detected at an early stage, have a high risk of causing symptoms.

Our study demonstrated extremely high ORs relating to female sex (7.2 adjusted) and breech position (24 adjusted) compared to that traditionally quoted in the literature. There may be an element of selection bias responsible for the latter, due to those in breech position routinely undergoing US screening; however, the wide confidence intervals suggest that chance may also be a factor.

### Other risk factors and theories of aetiology

Our statistical analysis shows that the second born child has an increased risk of DDH—less than the first born, but still higher than the risk for subsequent children. This has been found before but not widely publicised [6, 15].

‘Post-maturity’ has previously been suggested as a potential risk factor for DDH [10, 11, 13]. Our study confirmed that babies born after 38 weeks gestational age were indeed at a higher risk.

Our study demonstrated a correlation between higher birth weight and a higher risk of DDH in a dose–response fashion. This has on occasion been demonstrated previously [14], although it has more often not been replicated in a number of smaller studies, probably due to a lack of statistical power.

These three ‘less conventional’ risk factors are all plausible if one accepts the theory that DDH is related to ‘packaging disorders’. This theory suggests that it is tight constrictive conditions in the womb that lead to malposition of the hips, and subsequent DDH [5]. There is evidence that the hip becomes particularly lax for a short perinatal window, which may be an adaption to allow delivery through the birth canal [16].

Maternal age had a significant trend effect on incidence, but the effect was complex. Mothers aged 20–25 years had the lowest incidence, with incidence rising with increasing age over 25 years, but there was also a slight increase for mothers aged <20 years. One could postulate that this may be due to relative immaturity of the birth canal in very young mothers, but it remains hard to explain the higher incidence in the older mothers.

### Difference in risk factors between the sexes

All the above risk factors remained significant when analysed for girls alone. Subgroup analysis of boys only showed significance for breech position, other (non-cephalic) position and family history. It failed to show significance for the first or second born child, post-maturity, maternal age or high birth weight; however, as boys were the far smaller group, it would be expected that this would give rise to a lack of statistical power. It is therefore probably unwise to draw strong conclusions from this subgroup analysis.

### Comparison with previous studies

Several studies have found increased risk with female sex, family history, and breech position [17]. Some have not shown these, presumably due to lack of power. A meta-analysis of 28 publications failed to show a significant increased risk for primiparity or oligohydramnios, but the findings may have been diluted by including all studies rather than all high-power studies [18]. The larger studies tend to find more risk factors, with narrower confidence intervals, which would be expected due to their larger statistical power [5]. Although not perfect, our data contained few missing or inconsistent data values, and our statistical power, as evidenced by the low *p* values, was considerable, and we therefore feel this adds weight to our findings.

### Discussion of caesarean section

Vaginal delivery and caesarean section have been investigated before with conflicting results, probably due to a failure to control for confounding variables with logistic regression analysis [14]. We found with crude ORs that caesarean section appeared to increase the risk of DDH, but there was significant interaction with breech presentation and other variables. When these were controlled for, caesarean section actually reduced the risk. In a large study of the South Australian population, the same reduction in risk for breech babies if delivered by caesarean section was found [10]. This does not prove causation between mode of delivery and risk of DDH. However, it appears plausible that a vulnerable hip is more likely to be stretched or become decentered during a traumatic passage through the birth canal, particularly if in the breech position.

### Theories of aetiology

Most authors cite two probable classes of causative factor in DDH. One set, alluded to above, comes under ‘packaging’ disorders with late gestational age, high birth weight, high-tone primiparous womb, and abnormal positioning belonging to this theory. Congenital talipes equinovarus and congenital muscular torticollis are thought to draw their association with DDH from the same set of causes [19, 20]. An increased risk from vaginal delivery compared to caesarean section may imply a potential for exacerbation of latent instability during a tight and traumatic passage of a breech baby through the birth canal. The other major factors in aetiology are probably heritable—ligamentous laxity, and a shallow acetabulum.

### Proposal to modify the screening programme by calculating risk

The risk to different babies varied by orders of magnitude according to the combinations of risk factors, particularly sex, family history and breech position. Thus, it may be possible from our figures to calculate a risk for each individual, with appropriate confidence interval, and to base US screening on this risk. Deciding the threshold would be the difficult part, and any proposed change would have to be audited prospectively as results are not entirely predictable.

It is worth re-iterating that any selective US screening programme relies on good clinical screening of the remainder of babies. Therefore, it is crucial if US is only used selectively, that training of midwives, health visitors, GPs and paediatricians is ongoing in the detection of risk factors and in the technique of neonatal examination.

## Conclusions

This study has reinforced what is known about certain risk factors for DDH and has lent weight to the existence of other, less known risk factors. The majority of risk factors are compatible with a model of DDH which involves at least one heritable element that is more strongly expressed in females, a normal perinatal tendency to increased laxity, and additional environmental factors in the womb and during birth that may push a vulnerable hip towards dysplasia, instability and dislocation. This epidemiological information may help inform health care planning and screening policy. A selective US screening policy, as long as it is based on sound clinical examination and risk-based referral for US, can diagnose the majority of cases of DDH [9], but a small proportion of cases with normal neonatal examinations and no risk factors will still go undetected.

## References

[1] Bache CE, Clegg J, Herron M (2002). Risk factors for developmental dysplasia of the hip: ultrasonographic findings in the neonatal period. J Pediatr Orthop B.

[2] Sewell MD, Rosendahl K, Eastwood DM (2009). Developmental dysplasia of the hip. BMJ.

[3] Committee on Quality Improvement (2000). Clinical practice guideline: early detection of DDH of the hip. Pediatrics.

[4] Perry DC, Tawfig SM, Roche A, Sharriff R, Garg NK, James LA (2010). The association between clubfoot and developmental dysplasia of the hip. J Bone Joint Surg Br.

[5] Chan A, McCaul K, Cundy P, Haan E, Byron-Scott R (1997). Perinatal risk factors for developmental dysplasia of the hip. Arch Dis Child Fetal Neonatal Ed.

[6] Yiv BC, Saidin R, Cundy PJ, Tgetgel JD, Aquilar J, McCaul KA (1997). Developmental dysplasia of the hip in South Australia in 1991: prevalence and risk factors. J Paediatr Child Health..

[7] Woodacre T, Carlisle G, Cox PJ (2015). The “Ischial Limb”: a landmark on anterior ultrasound scanning used to assess reduction in developmental dysplasia of the hip. J Pediatr Orthop B.

[8] Carlisle G, Woodacre T, Cox PJ (2014). Verification of hip reduction using anterior ultrasound scanning during Pavlik harness treatment of developmental dysplasia of the hip. J Orthop.

[9] Woodacre T, Dhadwal A, Ball T, Edwards C, Cox PJA (2014). The costs of late detection of developmental dysplasia of the hip. J Child Orthop.

[10] Bower C, Stanley FJ, Kricker A (1987). Congenital dislocation of the hip in Western Australia. A comparison of neonatally and post-neonatally diagnosed cases. Clin Orthop Relat Res.

[11] Shipman SA, Helfand M, Moyer VA, Yawn BP (2006). Screening for developmental dysplasia of the hip: a systematic literature review for the US Preventive Services Task Force. Pediatrics.

[12] Loder RT, Skopelja EN (2011) The Epidemiology and demographics of hip dysplasia. ISRN Orthop. Article ID 238607:46. doi:10.5402/2011/238607 10.5402/2011/238607 PMC4063216 24977057

[13] Clarke NM, Reading IC, Corbin C, Taylor CC, Bochmann T (2012). Twenty years experience of selective secondary ultrasound screening for congenital dislocation of the hip. Arch Dis Child.

[14] Patterson CC, Kernohan WG, Mollan RAB, Haugh PE, Trainor BP (1995). High incidence of congenital dislocation of the hip in Northern Ireland. Paediatr Perinat Epidemiol.

[15] Studer K, Williams N, Antoniou G, Gibson C, Scott H, Scheil WK, Foster BK, Cundy PJ (2016). Increase in late diagnosed developmental dysplasia of the hip in South Australia: risk factors, proposed solutions. Med J Aust.

[16] Ralis Z, McKibbin B (1973). Changes in shape of the human hip joint during its development and their relation to its stability. J Bone Joint Surg Br.

[17] De Hundt M, Vlemmix F, Bias JMJ, Hutton EK, de Groot CJ, Mol BWJ (2012). Risk factors for developmental dysplasia of the hip: a meta-analysis. Eur J Obstetr Gynecol Reprod Biol.

[18] Ortiz-Neira CL (2010). Validating the risk factors for developmental dysplasia of the hip. A meta-analysis. Pediatr Radiol.

[19] Minihane KP, Grayhack JJ, Simmons TD, Seshadri R, Wysocki RW, Sarwark JF (2008). Developmental dysplasia of the hip in infants with congenital muscular torticollis. Am J Orthop.

[20] Pagon RW, Choudry Q (2009). Neonatal foot deformities and their relationship to developmental dysplasia of the hip: an 11-year prospective, longitudinal observational study. J Bone Joint Surg Br.

